# Supplementary material for: Stockholm Paradigm in the Study of Influenza H1N1 Viruses: A New Approach to the Study of Zoonotic Risk Coupling Multiple Correspondence Analysis and Multi-Locus Phylogenies
Source: Viruses. 2025 Oct 8;17(10):1350. doi: 10.3390/v17101350 (PMC12568263; doi:10.3390/v17101350)
Supplement: Supplementary file 1 [file viruses-17-01350-s001.zip › viruses-3828043-supplementary.pdf]

Supplementary Material

Here we present the full explanation of the pre processing workflow applied in order to each the results presented for Capacity and Opportunity analysis.

Preprocessing of raw datasets

Strain Table

The raw version of this data was obtained in January 2023 through this link (Raw Strain Data IRD, Olson, 2022). The downloaded version of the Influenza A H1N1 strain information gathered in the BV-BRC database consisted of 18,045 rows and 21 columns after we manually deleted variables (originally it had 37 columns) not related to the Strain Name or the Genbank ID information for all eight genome segments.

Therefore the dataset that was included in the pre-processing execution, later entitled BVBRC_strain/BVBRC_strain_filtered.csv includes the columns below (Table S1):

Column	Description	Variable	Example
0	subtype	Influenza subtype	H1N1
1	2_pb1	Genbank ID for second genomic segment	CY188807
2	6_na	Genbank ID for sixth genomic segment	CY188803
3	8_ns	Genbank ID for eighth genomic segment	CY188805
4	5_np	Genbank ID for fifth genomic segment	CY188804
5	1_pb2	Genbank ID for first genomic segment	CY188808
6	host_common_name	Host common name	Avian
7	isolation_country	Isolation Country	USA
8	7_mp	Genbank ID for seventh genomic segment	CY188802
9	host_name	Host Name	swine
10	taxon_id	Taxon identification in IRD bank	1523128
11	3_pa	Genbank ID for third genomic segment	CY188806
12	strain	Strain Identification	A/New York/WC-LVD-14-047/2014

13	genbank_accessions	All segment Genbank IDs	CY188801;CY188806;CY188807;CY188804;CY188802;CY1
14	geographic_group	Continent	North America
15	host_group	Host group	Nonhuman Mammal
16	collection_year	Year of collection	2014
17	n_type	Neuraminidase type	1
18	4_ha	Genbank ID for fourth genomic segment	CY188801
19	segment_count	Genomic segment number	8
20	h_type	Hemagglutinin type	1

Table S1. Strain table containing the Genbank ID for the 8 genome segments, the Strain Name, host information (common name and group), Hemagglutinin and Neuraminidase type as well as isolation country and geographic group.

Epitope Table

This dataset includes information on all linear peptides registered in the BV-BRC (or previous IRD) Epitope platform. Apart from all other datasets used in this work, this table does not have a column with the Strain Name. In this case, it was necessary to use the *Genbank ID* in order to collect the aminoacidic sequence, and then scan each of the eight aminoacid sequences for all strains for epitope presence.

The raw Epitope table was manually filtered so that it included only complete and unique (not present in other datasets) data information. Therefore, we edited the original table *BVBRC_epitope.csv* so that it only included, for the 8,595 epitopes, the following 13 parameters (Table S2):

Column	Description	Variable	Example
0	Epitope ID	Epitope number in BV-BRC	10003
1	Epitope Type	Epitope type	Linear peptide
2	Epitope Sequence	Aminoacidic sequence	DRLFFKCI
3	Organism	Species	Influenza A virus
4	Protein Name	Protein name	Matrix protein 2
5	Protein ID	Protein ID originally pulled from UniProtKB/Swiss-Prot database	P06821.1
6	Protein Accession	Protein accession code originally pulled from UniProtKB/Swiss-Prot databases	P06821
7	Start	Aminoacidic position in protein sequence	44.0
8	End	Aminoacidic position in protein sequence	51.0
9	Total Assays	Total epitope identification assays	2
10	Bcell Assays	Epitope identification assays with B cells	NaN
11	Tcell Assays	Epitope identification assays with B cells	0/3
12	Comments	Additional information	A/Short text

Table S2. Epitope dataset containing epitope ID in the IRD, epitope identification by different lymphocyte assays, epitope structure and sequence.

Substitutions Table

The fourth raw dataset includes the original data registered in the database that correspond to genomic substitutions that have been identified by published works and are described as substitutions that alter viral capacity to interact with their host (published in PubMed). For this dataset, we downloaded the substitutions that were divided into Human (Table S3), Avian (Table S4) and Mammalian (Table S5) substitution tables, all of which have similar structure, with the first column containing the Strain Name, whereas the remaining columns describe **presence, absence** or **unknown** information on substitution detection in the respective strain.

Human Substitutions Table:

Column	Description	Variable	Example
0	Strain Name	Viral strain name	A/England/257/2009
1	Subtype	Influenza A subtype	H1N1
2	Collection Date	Collection Date	05/09/2009
3	State / Province	Collection region	Influenza A virus
4	Country	Collection country	USA
5	Host	Host group	Human
6-34	Phenotypic substitutions	-	

Table S3. Human substitution dataset containing the Strain Name, Influenza subtype H1N1, sample location and time it was collected, as well as the genomic substitution columns.

Avian Substitutions Table:

Column	Description	Variable	Example
0	Strain Name	Viral strain name	A/England/257/2009
1	Subtype	Influenza A subtype	H1N1
2	Collection Date	Collection Date	05/09/2009
3	State / Province	Collection region	Influenza A virus
4	Country	Collection country	USA
5	Host	Host group	Human
6-31	Phenotypic substitutions	-	

Table S4. Avian substitution dataset containing the Strain Name, Influenza subtype H1N1, sample location and time it was collected, as well as the genomic substitution columns.

Mammalian (non-human) Substitutions Table:

Column	Description	Variable	Example
0	Strain Name	Viral strain name	A/England/257/2009
1	Subtype	Influenza A subtype	H1N1
2	Collection Date	Collection Date	05/09/2009
3	State / Province	Collection region	Influenza A virus
4	Country	Collection country	USA
5	Host	Host group	Human
6-31	Phenotypic substitutions	-	

Table S5. Non-human mammalian substitution dataset containing the Strain Name, Influenza subtype H1N1, sample location and time it was collected, as well as the genomic substitution columns.

Surveillance Table

This dataset includes all information on data collection (date and location in terms of coordinates, city, state/province and country), and host identification and condition (in terms of natural state, capture mode and health at the time of sampling). The raw data (*BVBRC_surveillance.csv*) has 96 columns, many of which are incomplete, incorrectly filled or completely empty. Therefore, we manually deleted those columns, and ended up with a modified raw table described below, later entitled *BVBRC_surveillance_filtered.csv* (Table S6).

Column	Description	Variable	Example
0	S	Sample number	STP_2020_1527
1	Sequence Accession	Genbank ID for all genomic segments	CY168429,CY168430,CY168427,CY168428,CY168423,CY168424,
2	Sample Material	Sample type	CY188803
3	Collection Year	Year of collection	2016
4	Collection Country	Collection country	USA
5	Collection State Province	Collection region	Wisconsin

6	Collection City	City of collection	Cahuil
7	Collection Latitude	Collection Latitude	-34.47928
8	Collection Longitude	Collection Longitude	-72.02064
9	Pathogen Test Type	Viral detection test type	Influenza A virus
10	Pathogen Test Result	Viral detection test result	Positive
11	Subtype	Influenza A subtype	H3N2
12	Strain	Strain Identification	A/Green-winged Teal/Wisconsin/08OS2292/2008(H3N2)
13	Host Identifier	Host ID in IRD	UGAI14-2125
14	Host Species	Specific taxon	Gallus gallus domesticus
15	Host Common Name	Host Common Name	Domestic Chicken
16	Host Group	Host Group	Avian
17	Host Sex	Host Sex	Female
18	Host Natural State	Host Natural State	Domestic
19	Host Capture Status	Host capture strategy	Active surveillance (e.g. trap)
20	Host Health	Host's condition at time of sampling	Healthy
21	Symptoms	Notable symptom	Temperature:101.6
22	Onset Hours	Time of initial symptoms	cough:4
23	Sudden Onset	Undetermined information	-

24	Diagnosis	Undetermined information	-
25	Pre Visit Medication	Undetermined information	-
26	Treatment	Undetermined information	-
27	Initiation Of Treatment	Undetermined information	-
28	Duration of Treatment	Undetermined information	-
29	Treatment Dosage	Undetermined information	-
30	Vaccination Type	Undetermined information	-
31	Days Elapsed to Vaccination	Undetermined information	-
32	Source of Vaccine Information	Undetermined information	-
33	Vaccine Lot Number	Undetermined information	-
34	Vaccine Manufacturer	Undetermined information	-
35	Vaccine Dosage	Undetermined information	-
36	Other Vaccinations	Undetermined information	-
37	Additional Metadata	Undetermined information	-
38	Comments	Undetermined information	-
39-79	Undetermined information	-	

Table S6. Surveillance dataset containing columns relative to host identification, geographic location and capture context, as well as host symptoms and outcome vaccination information and treatment attempts. Unfortunately most of the columns were incorrectly filled or empty.

This was then imported into the Surveillance data processing script as *BVBRC_surveillance_filtered.csv*.

After concluding the pre-processing application here described, we imported the respective data to the subsequent processing steps (Figure 1):

1. Strain Table was saved as *BVBRC_strain_filtered.csv* and imported into *Strain data processing.ipynb;*

2. Surveillance Table was saved as *BVBRC_surveillance_filtered.csv* and imported into *Surveillance data processing.ipynb;*

3. Epitope Table was saved as *BVBRC_epitope_filtered.csv* and imported into *Epitope_search.ipynb* and *Occurence_table.ipynb;*

4. Substitutions Tables were saved as *Avian_pheno_subs.csv*, *human_pheno_subs.csv,* and *mammal_pheno_subs.csv* and imported into *Capacity data processing and merging.ipynb*.

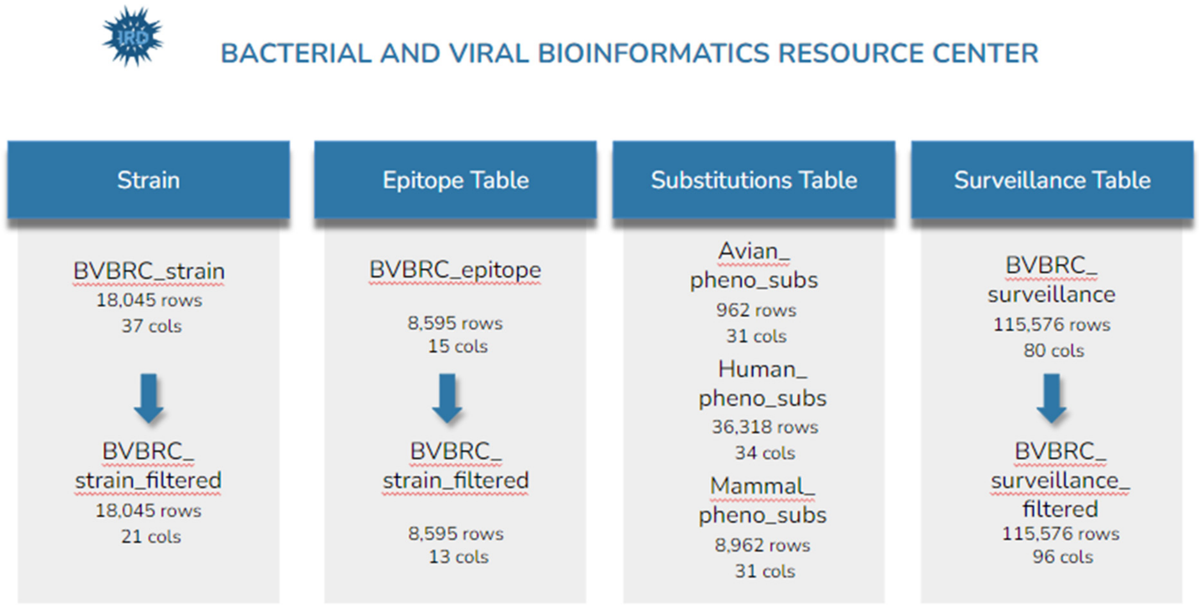

Figure S1. Summary of the preprocessing results

Data Processing protocol

After organizing all data in the previous step, we then import the preprocessed tables in order to generate a single table that contains all capacity (Strain, Epitope and Substitutions tables). The idea with this process is to construct a full dataset that does not contain empty or incorrectly filled cells, that is, all information must be boolean in nature, a basic requirement for the Multiple Correspondence Analysis (MCA). Moreover, each column should contain capacity information, whereas each row should represent a Strain, a structure also necessary for the MCA.

In order to reach this final goal, we started by importing each pre-processed table in its respective script as well as all necessary libraries, and then extracting only the columns of interest to this work, as will be described below (method workflow is described in Fig. 1 of Data Processing Protocol).

a. Strain Data Processing

The original Strain dataset contains 21 columns (described in the previous section *Data description*) which contains the subtype characterization, the strain name, host, collection and genome information. By analyzing this table along with the remaining three, we realized that host and collection information were also described in the Surveillance table, and therefore could be excluded from this dataset to avoid data redundancy. For the same reason we deleted the column *genbank_accessions*, since the same information was separated in the segment ID columns (i.e, 1_pb2).

The column **strain** was kept for this and all other datasets, and was used as the common column for all merging applications in the subsequent steps. By the end of this process, we turn the Strain Table (18,045 x 21) into *Strain_code_marge.csv*, with 18,045 rows and 9 columns.

b. Epitope Data Processing

The epitope information collected from the IRD does not carry *Strain Name* information. The pre-processed table has **Epitope ID** as the sampling unit, where each row brings the information for a single linear peptide sequence that is recognized by the immune system, according to previous works who utilized the IRD platform.

In order to organize this data so that it is readable for the MCA, it was required :

1. To obtain the aminoacidic sequence for each of the 8 genome segments of each of the 18,041 strains (common to all four databases). In order to do that we needed to (1) gather the *Genbank ID* of each segment of each strain (obtained from the Strain Table); (2) search and save the sequence associated to the *Genbank ID*; (3) translate it into aminoacidic sequence and (4) compress and save each of the translated sequences. This first process is referenced as *Epitope_search.ipynb.*

2. Generate the Epitope Occurrence table for each genome segment of all strains, where we import each of the 8 aminoacidic sequences of each strain and locate if an *Epitope sequence* (i.e, DSQTATKRIRMAIN) is present in either one of them. If present for the **x** strain's segment, the specific *Epitope sequence*'s cell will be filled with ***True***, whereas if not found, it will be filled with *False*. This process is repeated for all 8,595 epitope sequences and all 8 segments of all 18,041 strains. By the end of this process we have 8 epitope occurrence tables containing all substitutions for each of the genome segments.

   This process is referenced as *Occurence_table.ipynb*.

3. Analyze the Epitope frequency distribution in the database so that we could further filter epitopes considering their distinguishing power of strains. The idea of this second simple Exploratory analysis is to remove all epitopes that were present in less than 5% of all strains, in order to facilitate de dimensionality reduction by the MCA and avoid contributing to any possible noise the parameters might add to the hierarchical analysis.

For that end we constructed a frequency table for all epitopes present in the *merge_epi_compact.csv*, from which we analyzed the epitope and substitutions columns, corresponding with 7,371 adequate columns (that are informative, that is, present True/False variables), from the original 8,595 columns with capacity data.

The frequency table contained only two columns: the absolute (column 0) and relative frequency (column 1) from which we constructed a boxplot in order to collectively visualize the frequency distribution of all epitopes, and exclude those that were present in less than 5% of the 18,045 strains (Fig. 2). By the end of this application we ended up with 6,488 epitope-subs columns to be applied in the MCA (referred to as *Exploratory Epitope Analysis)*.

This process is referenced as *Epitope_table_processing.ipynb*.

	Abs Freq	Rel Freq
DSQTATKRIRMAIN	8950	0.496092
DTAQIIKL	8892	0.492877
DTAQIIKLL	8882	0.492323
DVIMEVVFPNEVGAR	8898	0.493210
EAIIVAMV	8911	0.493930
...	...	...
TNFLTEKAVASVKLAGNSSLCPIN	4510	0.249986
RQVIVERGNRSGYSGIF	4406	0.244221
SGLVGDTPRKNDSSSSSH	4441	0.246162
VNSDTVGWSWPDGAELPFTID	4494	0.249099
DRIPHRTLLMNELGV	4529	0.251039

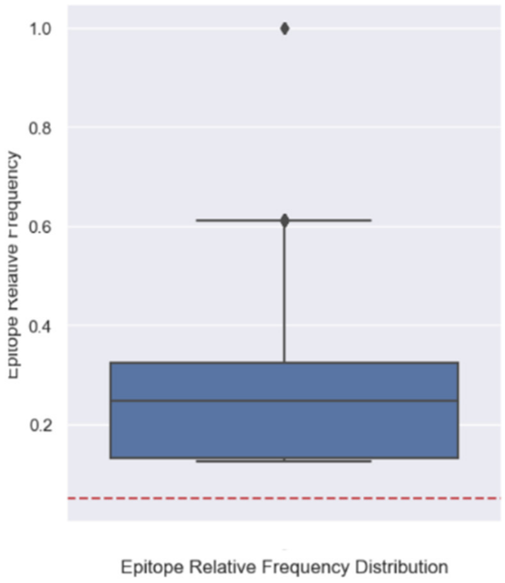

Figure S2. Frequency table and boxplot representing the frequency distribution of all 6,488 processed epitopes. The red dashed line signals the 5% epitope occurrence threshold, from which we decided to delete the two epitopes.

c. Substitutions Data Processing and Merging

Since our goal was to construct a final capacity table containing all datasets for all strains, we needed to also import and merge the Substitutions datasets, which are originally divided into three tables: Avian substitutions, Human substitutions and Mammal substitutions (Nonhuman markers). For these datasets we already had a *Strain Name* column, and thus simply required to be merged with the processed Epitope Table in order to generate a final capacity dataset using that column as the common denominator.

We preferred to do the merges separately, however, because we did not require that the *Strain Name* present in the Avian substitution table, for example, be present in the Human, Mammal. Therefore, we proceeded to merge *mammal substitutions* & *Epitope Table, human substitutions* & *Epitope Table* and *avian substitutions* & *Epitope Table,* in that order.

It is important to note that when we merged both epitope and substitution datasets, we identified the following Influenza strains host groups: of the 18,045 strains, 3,625 of them are non-human mammalian strains, 13,335 human and 608 avian strains, totalling 17,568 strains. The remaining strains were not present in the substitution table and were excluded from the analysis.

We then concatenate the resulting merges of Avian, Mammalian and Human substitutions, and create the final file *merge_epi_substitutions_09_11* which contains both **Epitope Table + Substitutions** data, for all three hosts. It this stage the final dataset 17,568 strains and 6,521 columns (as described in Fig. 3), which correspond to the cumulative capacity data and the strains that are present on both datasets (Genbank ID, epitope occurrence and substitution data), and is further processed in the *Final Table processing.ipynb* notebook in order to be checked for errors and formatting requirements before de MCA.

d. Final Table Processing

Before de MCA, we simply organized the capacity dataset so that all row indexes are defined by the *Strain Name* and delete all columns that will not be included in the

MCA, due to lack of information or error in data insertion. The Surveillance information will be included after the MCA application.

We also applied our Exploratory Data Analysis (EDA) (referred to Exploratory Capacity Analysis) to gather the general view of our final capacity dataset, for all columns. It is important to note that the EDA was also important to help us delete all columns that presented more or less than 2 variable types (something other than **True** or **False**), the first case being inadequate for the MCA application, since it only accepts boolean values, and the second being non-informative to help us distinguish the Influenza strains, possibly leading to noise in our analysis.

By the end of this application, we end up with the dataset containing all epitope and substitution data that presents only boolean variables, with the same dimension as described in the previous section.

All raw, pre-processed, processed data and final tables, as well as the scripts and development environment used in this work are available at DOI _______. All scripts can be accessed at: [Laboratorio-de-Analise-de-Dados/Influenza_Mestrado (github.com)].

e. Surveillance Data Processing

The Surveillance data required extra steps in order to reach the necessary format for the MCA, since its sampling unit was not, like the other datasets, the **Strain Name** but the **Sample ID**. We excluded all samples which were not identified by a strain name, and so 48,159 of the total 115,576 rows were used for the subsequent processes.

Among the 80 columns the filtered database contained, we were interested in all information that depicted any aspect of host interaction *opportunity*, or the ecological setting of the different Influenza strains. This dataset was the only one that actually approached this important aspect of host-parasite interaction, being spatio-temporal congruence among actual and potential hosts an essential aspect for new symbiont/interaction emergence or host-switching phenomena.

Considering the fact that this database was organized according to the Sample ID instead of the Strain Name, we had to apply a *spreading* technique which expands the rows present in one column and turns each row/variable into a new column/parameter. With this appliance, we were able to turn a *Collection country* column with **n variables** into **n columns** with each variable name. Each row would then contain **True/False** data, corresponding to the presence/absence of that Strain (now the row index) in that specific country.

The *spread* was applied to all information of potential interest, being Collection Year, Collection Country, Collection State, Collection City, Host Species, Host Common Name, Host Group and Host Natural State, as well as the original sampling unit *Sample ID* and coordinates - these last two variables were later excluded from the analysis since it did not contribute to our objective. One thing that was also required of us for this dataset was to edit the column *Strain* so that it did not present the subtype identified at the end of the strain name (i.e, A/Green-winged Teal/Wisconsin/08OS2292/2008(H3N2)), since for the remaining tables this information was not included.

We then stored this final structure, including Strain Name and Surveillance data, for the final cluster discussion, resulting in the two dataframes described in the Surveillance Data processing section (Host Nature Data and Host Location Data). Both datasets were then filed to be used as the *opportunity* information of the selected strains.

Additional Host Group Analysis

In order to add some depth into the data exploration of the Surveillance Information, we applied an additional method to identify Host Use, what we call Strain Name Segregation method. The idea here is to explore a larger number of strains that were not properly identified in the Surveillance Table, and did not reach the final processing step for that analysis (Surveillance Data Concatenation section).

Based on the three clusters identified by the HCA, we applied a simple method in

order to extract the host group the 17,568 strains were first identified - disclosed by their name tag (Figure S3). All Influenza strains are described according to the following name pattern:

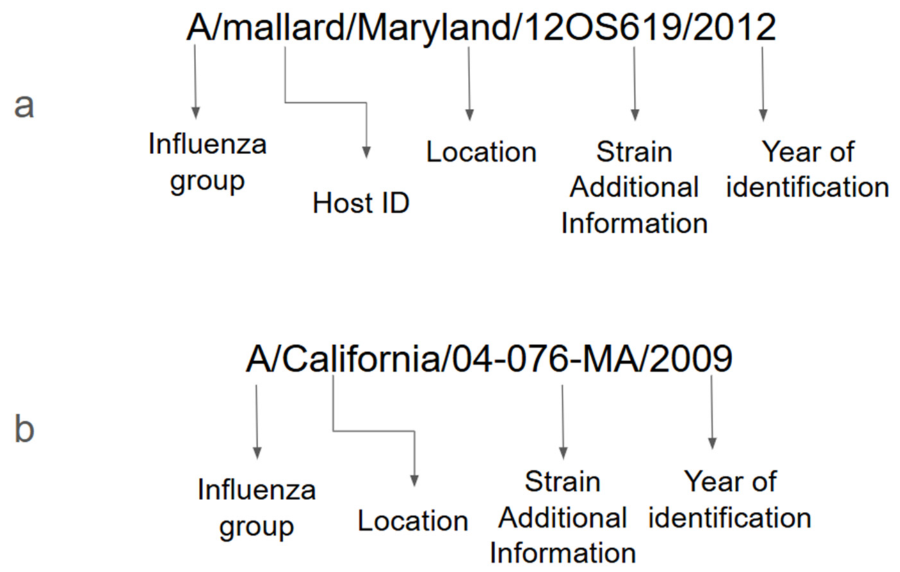

Figure S3. Description of strain name tag. ***a***. Describes the animal strain names, where the second term mentions the host name and the third describes its location (with no scale standardization). ***b***. Describes the human strain names, where the second term carries the Location information and there is no host name specification.

It is important to note here that this additional method is applied using the *crosstab* files, the outputs of the spreading technique described in the Supplementary Material. This application allowed us to observe the strain distribution across Host Groups for 4,210 strains, but does not enable a bigger discussion around the other Surveillance information as does the results presented in the main section of this work.

This method and application is described in the *Host Group Additional Analysis.ipynb* available in our GitHub repository.

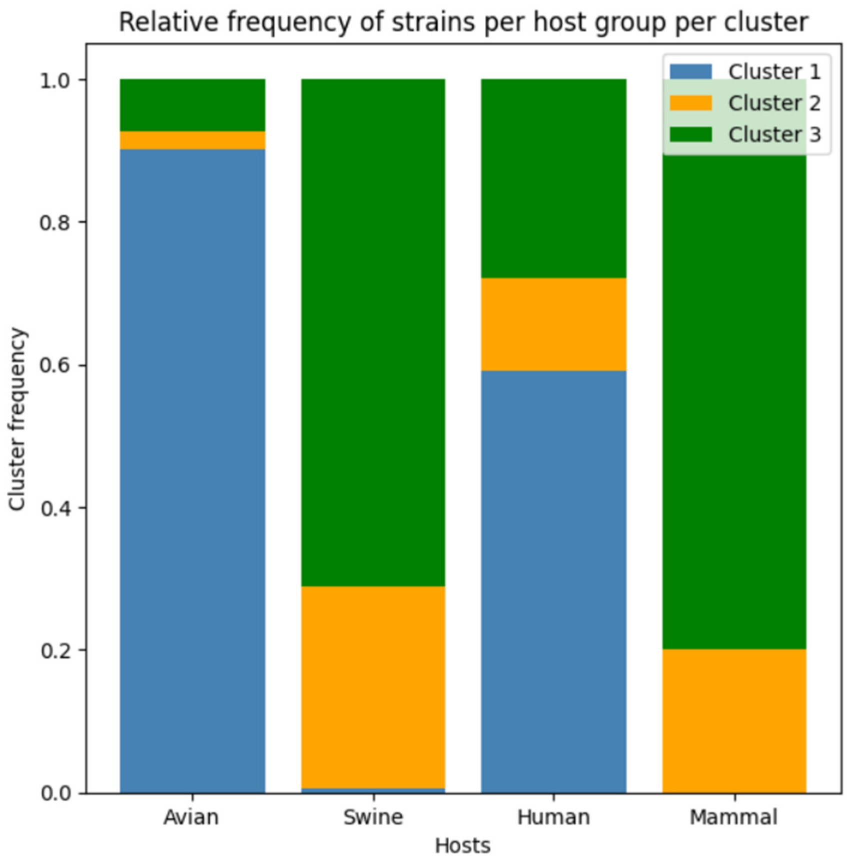

Figure S4. Relative frequency of strains per host group per cluster.

Unlike the main results, this dataset presented other Host Groups (Figure S4) - camels, cats and dogs - grouped by the Mammal tag. We can see here that the proportion of swine strains is altered compared to the main Surveillance Analysis, where the majority of strains that use this host are present in cluster 3, the majority of avian strains are from cluster 1 (also being the majority of cases in general - 2,565 of the 4,207 cases) and the majority of human strains are also from cluster 1.

Cluster 2 includes strains that mostly use Swine hosts (389 of the total 459 cluster 2 strains analysed here), and the mammalian cases cannot be discussed here since it includes only 10 strains - 8 belonging to cluster 3 and 2 belonging to cluster 2 (Figure S4).
